# *Helicobacter pylori* Status May Differentiate Two Distinct Pathways of Gastric Adenocarcinoma Carcinogenesis

**DOI:** 10.3390/curroncol30090578

**Published:** 2023-08-29

**Authors:** Martin Tobi, Douglas Weinstein, Mijin Kim, James Hatfield, Paula Sochacki, Edi Levi, Teisa An, Merlin Hamre, Vasundhara Tolia, Suzanne Fligiel, Rama Marepally, Jason Hallman, Bharati Bapat, Mei Yuan, Benita McVicker, Steven Gallinger

**Affiliations:** 1 Department of Research and Development, John D. Dingell VAMC, Detroit, MI 48201, USA; 2 Capital Health Medical Group, 2 Capital Way, Pennington, NJ 08534, USA; 3 Gastroenterology Division, University of Pennsylvania, Philadelphia, PA 19104, USA; 4 Department of Pathology, John D. Dingell VAMC, Detroit, MI 48201, USAsfligiel@umich.edu (S.F.); 5 Department of Pathology, Wayne State University School of Medicine, Detroit, MI 48201, USA; 6 Department Pediatrics, Children’s Hospital, Detroit, MI 48201, USA; 7 Department of Medicine, Mt Sinai Hospital, Toronto, ON N5T 3H7, Canada; 8 Division of General Surgery, Institute of Basic Medical Science of PLA Hospital, Beijing 100853, China; 9 Omaha Nebraska VAMC, Omaha, NE 68108, USA; 10 Hepatobiliary/Pancreatic Surgical Oncology Program, University Health Network, Toronto, ON M5G 2M9, Canada

**Keywords:** *H. pylori*, Adnab-9, monoclonal antibody, familial gastric cancer, FERAD ratio

## Abstract

Background: We evaluated the phenotype of sporadic gastric cancer based on HP status and binding of a tumor risk marker monoclonal, Adnab-9. Methods: We compared a familial GC kindred with an extremely aggressive phenotype to HP-positive (HP+) and -negative (HP−) sporadic gastric adenocarcinoma (GC) patients in the same community to determine if similar phenotypes exist. This might facilitate gene discovery to understand the pathogenesis of aggressive GC phenotypes, particularly with publications implicating immune-related gene-based signatures, and the development of techniques to gauge the stance of the innate immune system (InImS), such as the FERAD ratio (blood ferritin:fecal Adnab-9 binding OD-background binding). Resection specimens for the sporadic and familial group were stained for HP and examined for intestinal metaplasia (IM) and immunostaining for Adnab-9. Familial kindred specimens were also tested for the E-cadherin mutation and APC (adenomatous polyposis coli). Survival was evaluated. Results: Of 40 GC patients, 25% were HP+ with a greater proportion of intestinal metaplasia (IM) and gastric atrophy than the HP− group. The proband of the familial GC kindred, a 32-year-old mother with fatal GC, was survived by 13-year-old identical twins. Twin #1 was HP− with IM and Twin #2 was HP+. Both twins subsequently died of GC within two years. The twins did not have APC or E-cadherin mutations. The mean overall survival in the HP+ sporadic GC group was 2.47 ± 2.58 years and was 0.57 ± 0.60 years in the HP− group (*p* = 0.01). Survival in the kindred was 0.22 ± 0.24 years. Adnab-9 labeling was positive in fixed tissues of 50% of non-familial GC patients and in gastric tissue extract from Twin #2. The FERAD ratio was determined separately in six prospectively followed patient groups (*n* = 458) and was significantly lower in the gastric cancer patients (*n* = 10) and patients with stomach conditions predisposing them to GC (*n* = 214), compared to controls (*n* = 234 patients at increased risk for colorectal cancer but without cancer), suggesting a failure of the InImS. Conclusion: The HP+ sporadic GC group appears to proceed through a sequence of HP infection, IM and atrophy before cancer supervenes, and the HP− phenotype appear to omit this sequence. The familial cases may represent a subset with both features, but the natural history strongly resembles that of the HP− group. Two different paths of carcinogenesis may exist locally for sporadic GC. The InImS may also be implicated in prognosis. Identifying these patients will allow for treatment stratification and early diagnosis to improve GC survival.

## 1. Introduction

Premalignant markers accumulate with the progression of a gastric adenocarcinoma (GC) to cancer. Thus, most authorities agree that well-differentiated cancer arising in gastric adenomas progress through this sequence. Underlying genetic changes have been identified in GC, but the sequence progression is not as clear-cut for GC as it is for colorectal cancer (CRC), where most early changes can be characterized by progressive abnormal protein marker expression. Furthermore, there is controversy whether non-well-differentiated gastric cancer proceeds through an adenoma–carcinoma sequence. However, there is general agreement that H. pylori-associated GC arises in an environment of morphological change manifested first by antral chronic inflammation, then atrophy and, finally, by intestinal metaplasia (IM), but with the decline of H. pylori infection in Japan, non-H. pylori pathologic GC phenotypes are coming under scrutiny [1]. The morphological changes of IM have been well characterized and often contain all the cellular populations of small bowel mucosa, including Paneth cells. Two decades earlier, it was postulated that these changes are caused by chronic *Helicobacter pylori* (HP) infection and its association with GC, earning for *H. pylori* the title of a class I carcinogen in 1994 [2]. This theory is limited to distal GC and, disease of the cardia is considered to be distinct in its causation, thought to be associated with chronic gastroesophageal reflux disease (GERD). However, it remains debatable whether HP is an initiator of GC or only an association with distal cancers. Although the mortality from GC disease has somewhat decreased in the last decade, rates in the US for cancer of the gastric cardia have risen in the decade after HP discovery and may pose a major health risk to aging Americans if this trend continues [3]. Recent evidence shows that there is an impact of H. pylori to reduce the response of the innate and adaptive immune systems that may result in the modulation of GC-associated pathways [4]. Accordingly, there is still an urgent need for an improved non-invasive screening method and for studies to investigate the tumorigenesis of proximal gastric cancer through seeking common markers [5]. GC is a disease now considered to be theoretically preventable via treatment of HP infection, reversal of IM using anti-oxidants, and intensive treatment of GERD. However, these preventive measures remain experimental for the general high-risk population. There is an urgent need to develop surrogate markers to determine the response to risk reduction treatment within months, rather than several years, as is currently the case.

The Adnab-9 monoclonal antibody (MAb) was raised against the membranes of the earliest phenotypic lesion in colorectal neoplasia, the tubular adenoma. An association with a premalignant colon phenotype has been demonstrated [6,7,8]. Adnab-9, expressed in Paneth cells, is associated with the malignant phenotype in both CRC [7] and small intestinal carcinoma [9]. Among the initial serum markers available for the diagnosis of gastric cancer are CEA (carcinoembryonic antigen/CEACAM5), CA72-4 and CA19-9, but they lack sensitivity and specificity [10,11]. Inflammatory marker research suggested that IL-6 and a proteomic assay could have prognostic and diagnostic significance, but these also suffer from low sensitivity and specificity [12,13]. Another early proteomic assay showed prognostic significance for two markers, but these were only prognostic when under-expressed, and they both are non-specific [14]. Recently, non-invasive liquid biopsy has shown promise for GC diagnosis as a proxy for pathology biopsy and has numerous targets such as circulating tumor cells, tumor DNA and exosomes [15].

IM is considered a strong concomitant of gastric cancer and was found even in the pre-H. pylori era to frequently contain fully differentiated or immature Paneth cells, which may be important for proliferation in the gastric mucosa [16]. The Adnab-9 monoclonal that recognizes these cells has been shown to be diagnostic for gastric cancer through demonstrating reactivity in gastric juice, stool and premalignant tissue sections, regardless of the anatomical location of the gastric cancer [17]. This finding carries an implication of mucosal atrophy [18].

We had the opportunity to study this antigen within a gastric cancer kindred that included two 13-year-old identical twin boys that had an extremely aggressive biological course. In line with our study aim to seek out members of the community at high risk and validate the role of genetic changes and “H pylori”, the familial kindred phenotype was contrasted with an unselected patient population from the same geographic locale. Since GC is relatively uncommon in the US compared with the Far East, it did take some time to assemble the community data. We studied current familial gastric cancer markers, HP status, Adnab-9 binding and demographic markers and concluded that a gastric cancer phenotype with absent HP and negative Adnab-9 staining has an extremely aggressive phenotype.

## 2. Materials and Methods

### 2.1. Gastric Cancer Kindred

A 32-year-old African American female died of metastatic gastric cancer within 1 month of the onset of symptoms. She was survived by her identical twin boys, aged 13, and a maternal half-brother to the twins, aged 10. An endoscopic tumor biopsy of the mother, a gastric resection specimen from Twin #1 and stool and endoscopic biopsies of antral dysplastic polypoid lesions and the colon in the Twin #2 were available. Within a year of the mother’s death, Twin #1 was found to have iron deficiency anemia; gastroscopy showed gastric cancer, and he died shortly after resection. Twin #2 underwent surveillance endoscopy, and a gastric adenoma was found and removed. Colonoscopy was normal and follow-up gastroscopy revealed no change. A CLO-test (Campylobacter-like organism urease test) for HP on both gastroscopic occasions was positive in Twin #2. A CT (computerized tomography) scan showed an enlarged celiac lymph node, but further diagnostic procedures were declined. Twin #2 died of widely metastatic GC within 6 months of the initial endoscopy. Screening gastroscopy and colonoscopy were negative in the half-brother.

### 2.2. Patient Specimens and Analytical Methods

Forty-two sections were obtained from 40 patients diagnosed with gastric cancer at the Detroit VAMC (Veterans Administration Medical Center) from 1995 to 2000. Tissue specimens were stained with Giemsa or silver stains for HP testing. Urease testing via “CLO” tests on endoscopic biopsy specimens was also considered as positive evidence for HP infection. Sporadic gastric cancer patients were derived from the same location as the familial kindred twins. All tissue sections were fixed in 10% formalin and embedded in paraffin, and immunohistochemistry with Adnab-9 was performed on 4 μm sections as described previously [7,8,17,19]. Monoclonal anti E-cadherin labeling was kindly arranged by Dr. M.E. Key, and staining was performed at DakoCytomation Inc. (Carpinteria, CA, USA). The staining intensity was scored as +: 5–25%; ++: 25–50%; +++: >50%; and <5% was considered as negative [6]. A single pathologist (P.S.) reviewed all specimen histopathology. The diagnosis was based on clinical findings, laboratory tests, endoscopic examination and histopathology of endoscopic biopsy or surgically removed specimen. Gastric cancer patient details are summarized in Table 1.

Stool was available from a single twin (Twin #2) and contrasted with a blinded set of 10 stool samples (5 gastric cancer and 5 controls) obtained from the Department of Medicine, General Hospital of the Peoples Liberation Army (PLA) of Beijing, China. ELISA with Adnab-9 monoclonal antibody was performed with stool samples as previously described [17,20]. Normal-appearing gastric tissue was also available and extracted as described previously and contrasted with similar gastric tissue from an APC (adenomatous polyposis coli) patient [7,8].

Blood was obtained from Twin #2 and his half-brother for genetic testing. Peripheral blood lymphocyte DNA and RNA were extracted and analyzed for underlying germline APC mutation, which may cause FAP (familial adenomatous polyposis). Genetic assays for the APC gene (exons 2–5) were performed at the Lunenfield Cancer Centre Laboratory, Mt. Sinai Hospital (Toronto, ON, Canada) via both direct sequencing and in vitro synthesis of protein tests. Direct sequencing for both intronic and exonic E-cadherin was similarly performed for the entire molecule as previously described [21].

In order to establish the stance of the innate immune system based on the FERAD ratio (fecal Adnab-9 binding:blood ferritin), from our database, we ascertained 10 GC patients, 14 with CAG, 27 with IM, 17 with a GC family history in a first-degree relative, 156 with a past history of HP+ but no neoplasia on endoscopy and 234 HP-naïve patients via endoscopy and Hp serum or stool testing. A potential challenge was in the determination of the FERAD ratio as it has two components which can be altered by confounders such as medications, food supplements, anemia and contracting neoplastic disease. There were no differences in analysis we performed in terms of the above confounding factors, and the extensive follow-up allowed for surveillance for these conditions. We did not perform serum cancer antigen testing as serum was not available for most of the sporadic GC patients. Inclusion criteria were willingness to participate and being fit to have an endoscopic procedure, and conversely, exclusions were unwillingness to participate in the study and not being physically fit to have an endoscopic procedure. The time of follow-up was recorded.

This study met the standards of and was approved by the Detroit VAMC/WSU (Wayne State University, Detroit, MI, USA) IRB.

### 2.3. Statistical Evaluation

The data were analyzed using Student’s *t*-test and non-parametric methods of chi-square [22] and linear correlation using the method of least squares; survival data were censored and depicted using the method of Kaplan–Meier, and differences in survival were analyzed using the log rank test; multivariate analyses were also performed using a statistical computer software package (MedCalc Online; MedCalc Software Ltd., Ostend, Belgium). We used a pre-test online Kolmogorov–Smirnov calculation which can also be accessed online to check for normality for Student’s *t* testing and the Mann–Whitney non-parametric test where appropriate: https://www.socscistatistics.com/tests/kolmogorov/default.aspx, accessed on 4 April 2023. Any *p* values of <0.05 were considered significant.

### 2.4. Limitations

The major pitfalls stem from the extreme novelty of the familial kindred in that the affected members of the kindred were limited to 3 individuals. The literature also does not yield any comparable cases. Although were able to compare stool data, the available numbers were few. While we have considerable data in Chinese patients, in order to apply p87 assays to a local population with the same ethnicity as the kindred members, the latter were somewhat limited in number as distal disease in less common in the US, while proximal disease, which is not the focus of our study, is actually increasing [3,17].

## 3. Results

### 3.1. Adnab-9 Binding via ELISA in Kindred

**Figure 1:**
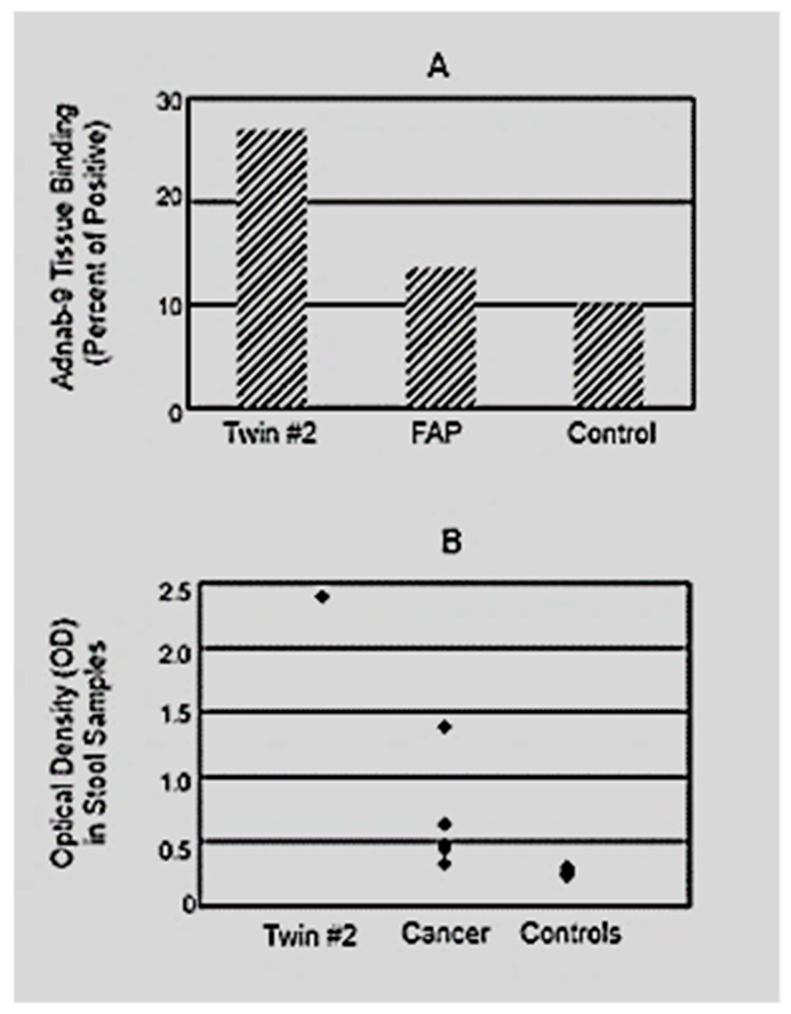
Adnab-9 binding via ELISA A. (**A**) bar diagram shows the Adnab-9 tissues binding in Twin #2 and APC patients. Results expressed in terms of percent of positive control in Adnab-9 binding. The Adnab-9 level standardized for protein was 2 times higher that of the APC patient and 2.7 times higher that of the control. (**B**) A scattergram shows the Adnab-9 stool ELISA in Twin #2 and Chinese gastric cancer patients. Results are expressed in terms of optical density. When the code was broken for the stool samples, the binding in specimens from cancer patients was greater than the controls; however, the level in Twin #2 was five times greater than the mean level of stools from the five patients with gastric cancer and much greater than five patients in the negative control group.

Fresh tissue from Twin #2 was available and compared to a normal gastric extract from endoscopic biopsies from a patient with APC with marked fundic polyposis and a normal control. The Adnab-9 level standardized for protein was 2 times higher in Twin #2 than that of the FAP patient and 2.7 times higher than that of the control when expressed in terms of percent of positive control in Adnab-9 binding (a membrane-enriched tissue extract of a colonic adenoma) and depicted in Figure 1A.

In view of the diagnostic ability for gastric cancer using Adnab-9 stool ELISA in Chinese patients [17], stool was obtained from Twin #2 and compared with those of five Chinese gastric cancer patients and five Chinese controls provided initially, and ELISA was performed in a blinded study as coded samples. When the code was broken on the stool samples, the level in Twin #2 was five times greater than the mean levels in patients with gastric cancer and even greater than in the negative controls (Figure 1B).

### 3.2. Genetic Testing for Germline APC (Adenomatous Polyposis Coli) Mutation

Familial adenomatous polyposis (FAP) is an autosomal dominant disorder which predisposes one to colon carcinoma and other cancers, including gastric. Germline APC (adenomatous polyposis coli) mutation is known to cause FAP [23]. The APC gene consists of 15 exons, and the majority of germline and somatic mutations occur between exons #3–15 [24]. We therefore tested for APC germline mutations in segments 2–5 (codons 686-2843) via in vitro synthesis of protein testing in Twin #2 and his half-brother. There were no APC germline mutations observed.

### 3.3. Tissue Testing Determination of H. pylori

**Figure 2:**
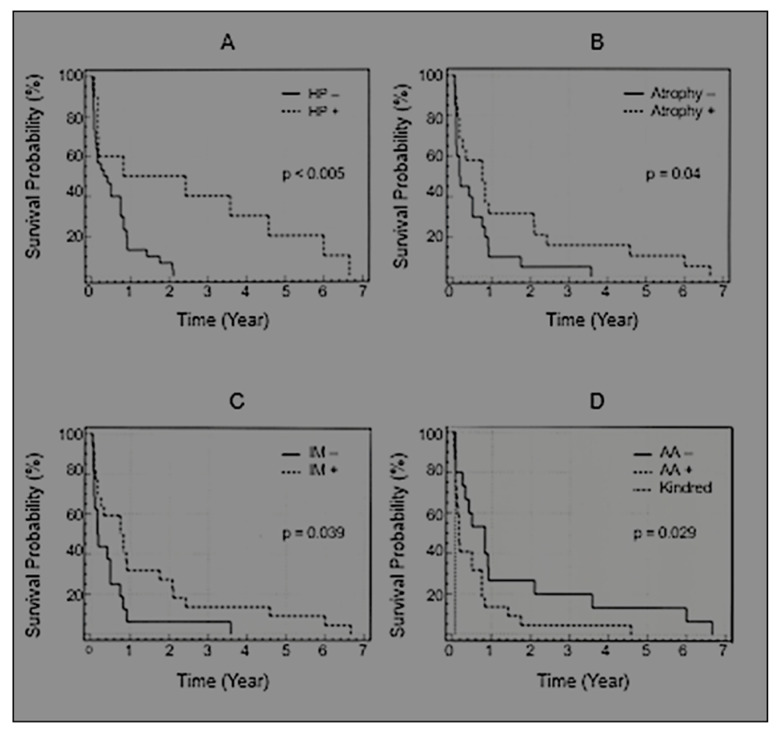
Kaplan–Meier curve depicts survival cases. (**A**) Overall survival was significantly longer in the HP+ group than in the HP− group (*p* < 0.005; CI 1.4–6.17); all above parameters analyzed via univariate testing using the log rank test). (**B**) Atrophy (*p* = 0.04); (**C**) metaplasia (0.039); (**D**) race (*p* = 0.029). (**A**–**D**) is depicted below showing Kaplan-Meier mortality curves for Hp status (**A**); gastric atrophy (**B**); (IM) intestinal metaplasia (**C**); and African Americans (AA—**D**).

Among 40 gastric cancer patients, 10 were HP+ and 30 were HP− as determined via tissue staining. The patient demographics are shown in Table 2. There was no difference between age (65.3 ± 14.6 years in HP+ and 64.6 ± 13.2 years in HP−; *p* = 0.54) or stage of disease (2.67 ± 1.22 in HP+ and 3.22 ± 1.18 in HP−; *p* = 0.5) in HP+ and HP− groups. Overall survival was significantly longer in the HP+ group than in the HP− group (*p* < 0.005; CI 1.4–6.17; all above univariate testing via log rank test), and Kaplan–Meier curve depictions are shown in Figure 1A. Multivariate analysis via Cox proportional-hazards regression analysis showed that HP+ status was an independent prognostic marker (*p* = 0.04). The mean survival in the HP+ sporadic GC group was 2.47 ± 2.58 and 0.57 ± 0.60 years in the HP− group (*p* = 0.01). Familial group survival was worse at 0.22 ± 0.24 years compared to the HP+ sporadic cases (*p* = 0.0049) and also, had a worse prognosis than HP− cases (*p* = 0.025). The age-adjusted survival in HP− sporadic patients was indeed similar to that of the kindred. The presence of IM and gastric atrophy tended to be higher in HP+ groups (70 versus 54% (NS) and 70% versus 43% (NS), respectively). In the non-familial gastric cancer group as a whole, there was a correlation between atrophy and metaplasia (*p* < 0.0001) as well as age and atrophy (*p* = 0.014). Other correlations with prognosis via univariate analysis with regard to morphological and demographic parameters were stage (*p* = 0.024), atrophy (Figure 2B, *p* = 0.04), metaplasia (Figure 2C, *p* = 0.039) and African American ethnic group status (*p* = 0.043). The latter was even more significant when also considering the familial group which has the same ethnic status (Figure 2D, *p* = 0.029). The average time for follow-up in years was 7.65.

**Table 1:** Cancer patient characteristics.

Category	Sub-Category	Number (*n* = 40)
Stage *	0	1
	I	4
	II	5
	III	6
	IV	20
Type *	Intestinal	20
	Diffuse	14
Differentiation	Well & Moderate	9
	Poor	17
	Signet cell Type	1
	Scirrhous/Mixed	1

**Figure 3:**
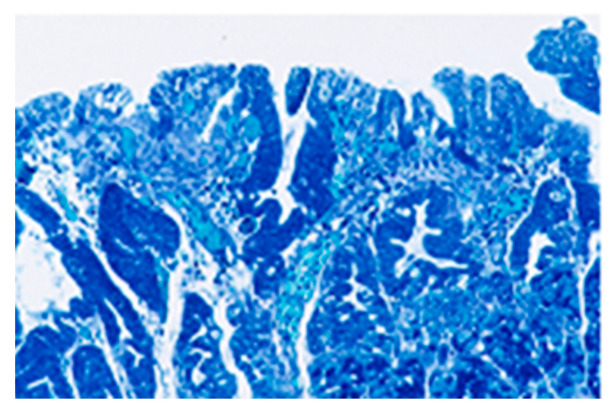
A silver stain of the gastric mucosa taken at surgery from Twin #1. Despite being HP-negative, there is marked intestinal metaplasia present. Magnification was 100×.

* Four patients were unclassifiable with regard to type, six for stage and twelve for differentiation. HP+ status was the only independent prognostic indicator as shown through multivariate analysis in the entire group (*p* = 0.032) or the non-familial as shown above. A silver stain for HP of the gastric mucosa taken at surgery from Twin #1 was negative but showed marked IM (Figure 3) while the CLO-test was positive for HP+ in Twin #2.

### 3.4. Tissue Labeling of Adnab-9 and E-cadherin in Gastric Tissue Sections

**Figure 4:**
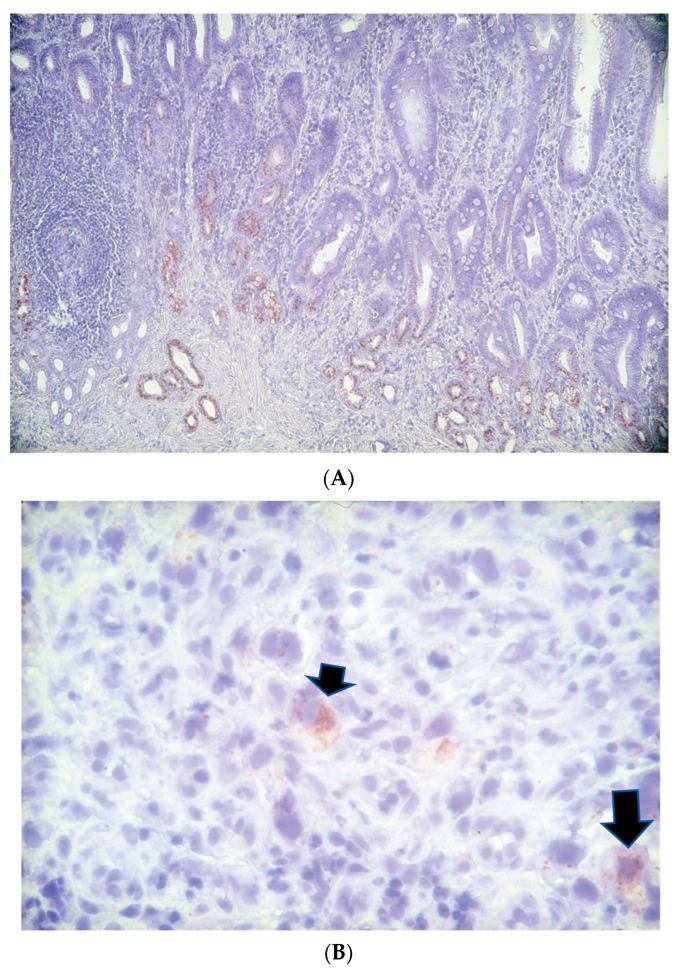
Adnab-9 labeling of sporadic gastric cancer. (**A**) This 70-year-old patient had HP + chronic atrophic gastritis and intestinal metaplasia with stage 4 intestinal-type gastric adenocarcinoma. Adnab-9 staining was 4+ in the deep glands in the vicinity of the cancer. The substrate was EAC, imparting a reddish-brown color. Magnification was 100×. (**B**) This 40-year-old patient had HP− chronic atrophic gastritis and stage 4 diffuse-type gastric adenocarcinoma. Adnab-9 staining was 3+ in the cytoplasm of very large cancer cells (arrows). The substrate was EAC, imparting a reddish-brown color. Magnification was 400×.

Tissue samples were available from 80% (32 out of 40) of sporadic GC patients at resection and were stained with Adnab-9 monoclonal antibody, an early neoplasia marker. Of these, 16 (50%) labeled positively, and about one third of cancerous tissues were positive (Figure 4A,B).

The univariate analysis showed that Adnab-9 labeling intensity in any tissue was positively correlated with survival (*p* < 0.034). In the kindred, one section from the mother’s diagnostic endoscopic biopsy was too small to be representative for labeling, and the sections from the twins were negative. This suggests that this unique family with an extremely aggressive form of GC had a genetic predisposition to GC.

**Figure 5:**
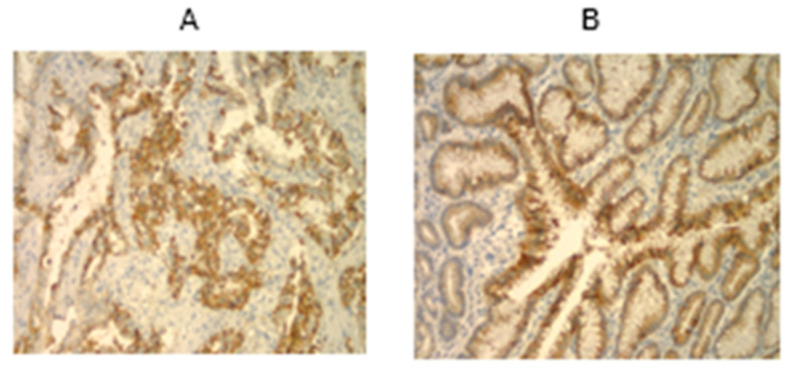
E-cadherin labeling in Twin #1 showed intense labeling in both the cancerous and corresponding benign tissue. (**A**) Diffuse cytoplasmic staining in low power of cancer tissue. The substrate was diaminobenzidine (DAB), imparting a golden-brown color. Magnification was ×75. (**B**) Membrane staining in hyperplastic glands in high power. This suggests that there was no mutation of E-cadherin in the kindred. The substrate was diaminobenzidine (DAB), imparting a golden-brown color. Magnification was ×100.

E-cadherin is a transmembrane protein and plays a critical role in establishing cell polarity, maintaining normal tissue morphology, and cellular differentiation [25]. Down-regulation of E-cadherin has been found in cancers including gastric cancer [26]. E-cadherin germline mutations have been observed in familial gastric cancer [27]. We therefore performed genetic testing of this gene. E-cadherin labeling in Twin #1 showed intense labeling, both in the cancerous and corresponding benign tissue (Figure 5), suggesting that there was no mutation of E-cadherin in the kindred.

### 3.5. Studies on the Innate Immune System as Expressed Using the FERAD Ratio and Demographics

Table 3 depicts demographics of the patient groups evaluated using FERAD ratios.

## 4. Discussion

Gastric cancer in the pediatric age group is rare and usually associated with recognized polyposis germline mutations (*APC* for familial adenomatosis polyposis coli and *STK11* for Peutz–Jeghers syndrome) and E-cadherin mutations in patients as young as 15 years old or younger [28]. However, these aforementioned mutations are not exclusively demonstrated in all cases, as demonstrated in our kindred. Some patients may have presented with immunodeficiency syndromes [27,29]; however, this was not evident in our kindred. Cases in identical twins have been described in adults but have not been described in children. One case of two singlet siblings has been reported and one did have invasive cancer while the other proved only to have a dysplastic lesion. Other cases have been reported after chemotherapy and radiation. Prognosis is usually dependent on the cancer stage and HP status. However, data on whether the latter marker is an independent prognostic marker was previously lacking. We therefore believe that our kindred is unique and lacks the above deletions predisposing them to GC. In the effort to provide some environmental context, we looked at a group of gastric cancer patients in the same community. In this group, the only independent prognostic modifier in the analysis was the presence of HP infection.

Controversy still exists regarding the association of HP with GC. A retrospective study of 128 patients, surgically resected for GC, showed a seroprevalence of 64% HP+ associated with localized distal cancer but was not correlated with invasion depth, lymph node spread, cell ploidy or type. While correlated with survival, HP+ was not an independent predictor of prognosis [30]. Furthermore, HP+ status did not correlate with other more controversial GC prognostic markers such as c-erb-B2 and p53 overexpression in 112 cases [31], and no effect of HP+ on these mutations in 42 cases was described in another study [32]. While HP has been postulated to explain the familial clustering of gastric cancer [32], in our kindred, active HP infection was not demonstrated in Twin #1, although he had evidence for IM (Figure 2). The presence of HP in Twin #2 may not be germane to this hypothesis as HP is an unlikely initiator in this patient due to his extreme youth.

The non-detection of APC mutation in the first segment (exons 2–5) of the gene is probably not the cause of GC in this family. A mutation in the first segment still remains a possibility despite the absence of colonic polyps or dysplasia in the second twin. In addition, there was no E-cadherin mutation in the kindred, and gastric expression was normal at the tissue level in both the normal and cancerous tissue.

In a variety of pancreatic cancer, Adnab-9 positive labeling has been associated with good prognosis in invasive intraductal papillary mucinous neoplasms (IPMNs) and sporadic pancreatic adenocarcinoma long-term survivors [33,34]. In the present study, labeling was not seen in the kindred, and this may correlate with their extremely poor prognosis. Adnab-9 labeling in the sporadic group did correlate with survival on univariate analysis. The numbers were too small to obtain meaningful multivariate analysis, and accordingly, Adnab-9 labeling did not prove to be an independent prognostic factor. In the kindred, Twin #2 did have a very high stool OD (optical density) for Adnab-9 binding (Figure 2), but we did not have sufficient cancer patient data to compare OD levels with the presence of neoplasia.

**Figure 6:**
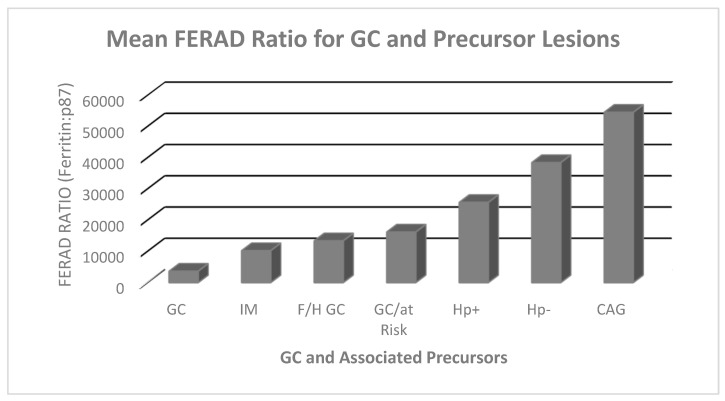
A bar diagram depicting FERAD ratios in each patient group. The premorbid FERAD ratios are the lowest for GC and tend to be less than either HP+ or HP− groups (*p* = 0.08; Mann–Whitney) as summarized in Table 3.

We have previously shown that Adnab-9 labels pre-malignant lesions of gastric origin and is detectable in stool, correlating with the presence of both gastric cancer and pre-malignant lesions [17], but this is the first report of the intensity of Adnab-9 staining correlating with prognosis. We may therefore consider Adnab-9 both as a diagnostic and potential prognostic marker for gastric cancer and a risk marker for the disease. The fecal level of Adnab-9 binding in the member of kindred was well in excess of the highest levels seen in patients from China, where the disease is endemic. Twin #2 did not have evidence of IM or atrophy to explain the high fecal levels, but this was the only cancer-associated marker we were able to identify in the kindred. We conclude that HP− disease, particularly in the African American community, is a prognostic factor for aggressive disease in univariate analysis, although mortality rates are not deemed to be different from US (United States) Caucasians [35]. However, AAs have a higher baseline prevalence of HP+ [18]. There are certainly dissenting voices in ascribing a less direct carcinogenetic role to HP and emphasizing the role of immune responsiveness [36], opposite to what we see in the low FERAD scores in GC patients, which may be the end result of a final common carcinogenetic pathway with impaired innate immunity. There have been relatively few studies focusing on HP− GC [37], and outcomes for mortality are rarely recorded. In that cited study, only 6.5% of GC cases were HP− (*n* = 55). The only associated risk factors appeared to be a twofold increase of both a family history of GC and current smoking. Although our numbers are somewhat smaller, our study is broader in scope and highlights the higher associated mortality and thus the importance of timely intervention. Recently, the latter was documented in a single-center, double-blinded trial [38] that showed that HP treatment in patients with a family history of GC in a first-degree relative can reduce GC by 55%. The authors concluded that the HP GC pathway is not a sequential adenoma–carcinoma pathway since there was no reduction of gastric adenomas, with about 8% incidence in both groups. In Figure 6, we show that the FERAD ratio is very low in GC patients but significantly elevated in patients with HP+, suggesting that GC is under the InImS radar, but HP+ status might mitigate a poor outcome. Recent work on GC has focused on innate genes, supporting our contention that immune-related gene-based signatures can be prognostic [39], and this has also been confirmed recently [40]. FERAD prognostic scores may be widely applicable as they have recently been validated in a COVID-19 study [41]. Technically, FERAD scores may be altered by dietary nutrients which may alter the carcinogenetic pathway, and we have suggested in the Introduction that intense treatment of GERD may be similarly effected in GC of the gastric cardia [42]. The latest paper looking at these markers singled out CEA as the most effective marker and confirmed that CA72-4 is outmoded [43]. Adnab-9 is one of the consistent front runners according to multiple reviews [44,45], so we did not attempt a direct comparison to CEA. Although we did not correlate stool p87/FERAD with immune checkpoint data, this may have important implications for GC interventions in that a TIME (tumor-immune microenvironment-exhausted phenotype) has been determined in GC that may be treated with immune checkpoint inhibitors such as pembrolizumab, approved for this use [46].

## 5. Conclusions

Our data suggest that a possible genetic basis for this phenomenon is likely, and its identification and subsequent use in screening could potentially help devise early diagnosis and ultimately prognosis in this community. Despite recent attention, the publication on the burden of GC disease in AAs has been slow in accrual. PubMed counts 52 publications from 1991 to 2022 (1.7/year) and an unexplained reduction in GC incidence between 2009–2018. The publication record may explain why the AA kindred described is unprecedented but [47] shows survival similarities with the Hp− group in terms of overall survival. It also explains why the FERAD data accrual process was so prolonged. The cancer phenotype with absent HP and negative Adnab-9 staining gastric cancer has an extremely aggressive phenotype (*p* < 0.034).

## Figures and Tables

**Table 2:** Patient demographics.

*Category*	Kindred (n = 3)	HP+ GC (n = 10)	HP− GC (n = 30)
Age (years) Age Range	20.7 ± 9.8 * (13–32)	63.5 ± 14.6 (29–80)	64.6 ± 13.2 * (40–89)
Atrophy		70% ^+^	43%
Intestinal Metaplasia	33%	70% ^@^	54%
Prognosis (years)	0.22 ± 0.24	2.47 ± 2.58 *	0.57 ± 0.6

* Stage was equally distributed in the HP+ and HP− groups. HP+ group survival correlated with stage (*p* < 0.05, r = 0.78), and survival/year life lived was similar in the HP− group and kindred, statistically lower than that of the HP+ group (*p* < 0.05). ^+^ OR4.85 (CI 1.08–21.84); *p* < 0.068; ^@^ OR4.38 (CI 0.99–19.27); *p* < 0.08; borderline.

**Table 3:** Demographics of the patient groups in Figure 3.

Demographic	Gastric Ca	CAG	Int Meta	F/H GC	HP+	HP−
Number (458)	10	14	27	17	156	234
Age (x yrs ± sd)	63.5 ± 12.7	61.8 ± 14.9	61.6 ± 14.8	66.3 ± 10.2 *	61.9 ± 11.1	56.3 ± 11.7
Race (%AA)	50	64	46	50	71	51
Gender (%M)	100	82	93	94	94	85
HP (%)	40	40	29	20	As defined	As defined
BMI	27.1 ± 2.8	31.5 ± 6.9	27.6 ± 4.8	28.2 ± 4.9	28.0 ± 4.7	28.6 ± 6.0
Mortality (%)	50	41.7	36.7	70.6	34	33

* *p* < 0.008 versus Hp—group; Ca = cancer; CAG—chronic active gastritis; Int Meta—intestinal metaplasia; F/H GC—family history of gastric cancer; HP+—*Helicobacter pylori* positive; HP−—*Helicobacter pylori* negative; x—mean.

## Data Availability

We aim to share data under Federal Government Guidelines.

## References

[1] Abe H., Ushiku T. (2022). Pathological Diversity of Gastric Cancer from the Viewpoint of Background Condition. Digestion.

[2] Suerbaum S., Michetti P. (2002). *Helicobacter pylori* Infection. N. Engl. J. Med..

[3] Corley D.A., Kubo A. (2004). Influence of Site Classification on Cancer Incidence Rates: An Analysis of Gastric Cardia Carcinomas. J. Natl. Cancer Inst..

[4] Khatoon J., Rai R.P., Prasad K.N. (2016). Role of *Helicobacter pylori* in Gastric Cancer: Updates. World J. Gastrointest. Oncol..

[5] So J.B.-Y., Yeoh K.-G., Moochala S., Chachlani N., Ho J., Wong W.-K., Mack P., Goh P.M.-Y. (2002). Serum Pepsinogen Levels in Gastric Cancer Patients and Their Relationship with *Helicobacter pylori* Infection: A Prospective Study. Gastric Cancer.

[6] Tobi M., Elitsur Y., Moyer M.P., Halline A., Deutsch M., Nochomovitz L., Luk G.D. (1993). Mucosal Origin and Shedding of an Early Colonic Tumor Marker Defined by Adnab-9 Monoclonal Antibody. Scand. J. Gastroenterol..

[7] Tobi M., Maliakkal B., Zitron I., Alousi M., Goo R., Nochomovitz L., Luk G. (1992). Adenoma-Derived Antibody, Adnab-9 Recognizes a Membranebound Glycoprotein in Colonic Tissue and Effluent Material from Patients with Colorectal Neoplasia. Cancer Lett..

[8] Tobi M., Maliakkal B.J., Alousi M.A., Voruganti V., Shafiuddin M., Yang S., Gesell M.S., An T., Hatfield J.S., Fligiel S. (1992). Cellular Distribution of a Colonic Adenoma-Associated Antigen as Defined by Monoclonal Antibody Adnab-9. Scand. J. Gastroenterol..

[9] Tobi M., Kaila V., Hassan N., Gallinger S., Fligiel S., Hatfield J., Gesell M., Sakr W., Luk G., Odze R.D. (1999). Monoclonal Antibody Adnab-9 Defines a Preneoplastic Marker in Epithelium at Risk for Adenocarcinoma of the Small Intestine. Hum. Pathol..

[10] Ellis D., Speirs C., Kingston R., Brookes V., Leonard J., Dykes P. (1978). Carcinoembryonic Antigen Levels in Advanced Gastric Carcinoma. Cancer.

[11] Kochi M., Fujii M., Kanamori N., Kaiga T., Kawakami T., Aizaki K., Kasahara M., Mochizuki F., Kasakura Y., Yamagata M. (2000). Evaluation of Serum Cea and Ca19-9 Levels as Prognostic Factors in Patients with Gastric Cancer. Gastric Cancer.

[12] Ashizawa T., Okada R., Suzuki Y., Takagi M., Yamazaki T., Sumi T., Aoki T., Ohnuma S., Aoki T. (2005). Clinical Significance of Interleukin-6 (Il-6) in the Spread of Gastric Cancer: Role of Il-6 as a Prognostic Factor. Gastric Cancer.

[13] Kon O.L., Yip T.-T., Ho M.F., Chan W.H., Wong W.K., Tan S.Y., Ng W.H., Kam S.Y., Eng A.K., Ho P. (2008). The Distinctive Gastric Fluid Proteome in Gastric Cancer Reveals a Multi-Biomarker Diagnostic Profile. BMC Med. Genom..

[14] Sousa J.F., Ham A.-J.L., Whitwell C., Nam K.T., Lee H.-J., Yang H.-K., Kim W.H., Zhang B., Li M., LaFleur B. (2012). Proteomic Profiling of Paraffin-Embedded Samples Identifies Metaplasia-Specific and Early-Stage Gastric Cancer Biomarkers. Am. J. Pathol..

[15] Zhang Z., Wu H., Chong W., Shang L., Jing C., Li L. (2022). Liquid Biopsy in Gastric Cancer: Predictive and Prognostic Biomarkers. Cell Death Dis..

[16] Albedi F., Lorenzetti E., Contini M., Nardi F. (1984). Immature Paneth Cells in Intestinal Metaplasia of Gastric Mucosa. Appl. Pathol..

[17] Qiao S.X., Yuan M., Liu Y.L., Lin X.S., Zhang X.P., Tobi M. (2003). Detection of Gastric Cancer and Premalignant Lesions by Novel Marker Glycoprotein 87 Using Monoclonal Antibody Adnab-9. Cancer Epidemiol. Biomark. Prev..

[18] Shah S.C., Piazuelo M.B., Kuipers E.J., Li D. (2021). Aga Clinical Practice Update on the Diagnosis and Management of Atrophic Gastritis: Expert Review. Gastroenterology.

[19] Hsu S.-M., Raine L., Fanger H. (1981). Use of Avidin-Biotin-Peroxidase Complex (Abc) in Immunoperoxidase Techniques: A Comparison between Abc and Unlabeled Antibody (Pap) Procedures. J. Histochem. Cytochem..

[20] Yuan M., Xhang X., Leu Y., Xu Y., Ullah N., Lawson M., Tobi M. (2006). Fecal Adnab-9 Binding as a Risk Marker for Colorectal Neoplasia. Cancer Lett..

[21] Guilford P., Hopkins J., Harraway J., McLeod M., McLeod N., Harawira P., Taite H., Scoular R., Miller A., Reeve A.E. (1998). E-Cadherin Germline Mutations in Familial Gastric Cancer. Nature.

[22] Ingram D., Bloch R.F., Ingram D. (1985). Mathematical Methods in Medicine, Statistical and Analytical Techniques. Handbook of Applicable Mathematics Part 1.

[23] Half E., Bercovich D., Rozen P. (2009). Familial Adenomatous Polyposis. Orphanet J. Rare Dis..

[24] Nagase H., Nakamura Y. (1993). Mutations of the Apc (Adenomatous Polyposis Coli) Gene. Hum. Mutat..

[25] Shimoyama Y., Hirohashi S. (1991). Expression of E-and P-Cadherin in Gastric Carcinomas. Cancer Res..

[26] Mayer B., Johnson J.P., Leitl F., Jauch K.W., Heiss M.M., Schildberg F.W., Birchmeier W., Funke I. (1993). E-Cadherin Expression in Primary and Metastatic Gastric Cancer: Down-Regulation Correlates with Cellular Dedifferentiation and Glandular Disintegration. Cancer Res..

[27] Grunwald G.B. (1993). The Structural and Functional Analysis of Cadherin Calcium-Dependent Cell Adhesion Molecules. Curr. Opin. Cell Biol..

[28] Deutsch F., Zilberstein B., Yagi O.K., Crescentini F., Deutsch C.R., Gama-Rodrigues J.J. (2004). Gastric Carcinoma in a 13-Year-Old Girl. Gastric Cancer.

[29] Lee W.J., Lin J.T., Shun C.T., Lee W.C., Yu S.C., Lee P.H., Chang K.J., Wei T.C., Chen K.M. (1995). Comparison between Resectable Gastric Adenocarcinomas Seropositive and Seronegative for *Helicobacter pylori*. Br. J. Surg..

[30] Shun C.-T., Wu M.-S., Lin J.-T., Chen S.-Y., Wang H.-P., Lee W.-J., Wang T.-H., Chuang S.-M. (1997). Relationship of P53 and C-Erbb-2 Expression to Histopathological Features, *Helicobacter pylori* Infection and Prognosis in Gastric Cancer. Hepato-Gastroenterology.

[31] Kubicka S., Claas C., Staab S., Kühnel F., Zender L., Trautwein C., Wagner S., Rudolph K.L., Manns M. (2002). P53 Mutation Pattern and Expression of C-Erbb2 and C-Met in Gastric Cancer: Relation to Histological Subtypes, *Helicobacter pylori* Infection, and Prognosis. Dig. Dis. Sci..

[32] Chang Y.W., Han Y.S., Lee D.K., Kim H.J., Lim H.S., Moon J.S., Dong S.H., Kim B.H., Lee J.I., Chang R. (2002). Role of *Helicobacter pylori* Infection among Offspring or Siblings of Gastric Cancer Patients. Int. J. Cancer.

[33] Tobi M., Hatfield J., Adsay V., Galagan K., Kozarek R., Inagaki M., Kasai S., Tokusashi Y., Obara T., Hruban R.H. (2001). Prognostic Significance of the Labeling of Adnab-9 in Pancreatic Intraductal Papillary Mucinous Neoplasms. Int. J. Pancreatol..

[34] Tobi M., Kim M., Weinstein D.H., Rambus M.A., Hatfield J., Adsay N.V., Levi E., Evans D., Lawson M.J., Fligiel S. (2013). Prospective Markers for Early Diagnosis and Prognosis of Sporadic Pancreatic Ductal Adenocarcinoma. Dig. Dis. Sci..

[35] Hundahl S.A., Phillips J.L., Menck H.R. (2000). The National Cancer Data Base Report on Poor Survival of US Gastric Carcinoma Patients Treated with Gastrectomy: American Joint Committee on Cancer Staging, Proximal Disease, and the Different Disease Hypothesis. Cancer.

[36] Graham D.Y., Zou W.Y. (2018). Guilt by Association: Intestinal Metaplasia Does Not Progress to Gastric Cancer. Curr. Opin. Gastroenterol..

[37] Morais S., Peleteiro B., Araújo N., Malekzadeh R., Ye W., Plymoth A., Tsugane S., Hidaka A., Hamada G.S., López-Carrillo L. (2022). Identifying the Profile of *Helicobacter pylori*–Negative Gastric Cancers: A Case-Only Analysis within the Stomach Cancer Pooling (Stop) Project. Cancer Epidemiol. Biomark. Prev..

[38] Choi I.J., Kim C.G., Lee J.Y., Kim Y.-I., Kook M.-C., Park B., Joo J. (2020). Family History of Gastric Cancer and *Helicobacter pylori* Treatment. N. Engl. J. Med..

[39] Qiu X.-T., Song Y.-C., Liu J., Wang Z.-M., Niu X., He J. (2020). Identification of an Immune-Related Gene-Based Signature to Predict Prognosis of Patients with Gastric Cancer. World J. Gastrointest. Oncol..

[40] Ji B., Qiao L., Zhai W. (2023). Cgb5, Inhba and Traj19 Hold Prognostic Potential as Immune Genes for Patients with Gastric Cancer. Dig. Dis. Sci..

[41] Tobi M., Bluth M.H., Rossi N.F., Demian E., Talwar H., Tobi Y.Y., Sochacki P., Levi E., Lawson M., McVicker B. (2023). In the SARS-Cov-2 Pandora Pandemic: Can the Stance of Premorbid Intestinal Innate Immune System as Measured by Fecal Adnab-9 Binding of P87: Blood Ferritin, Yielding the Ferad Ratio, Predict COVID-19 Susceptibility and Survival in a Prospective Population Database?. Int. J. Mol. Sci..

[42] Kim J.-J. (2013). Upper Gastrointestinal Cancer and Reflux Disease. J. Gastric Cancer.

[43] Liu H.-N., Yao C., Wang X.-F., Zhang N.-P., Chen Y.-J., Pan D., Zhao G.-P., Shen X.-Z., Wu H., Liu T.-T. (2023). Diagnostic and Economic Value of Carcinoembryonic Antigen, Carbohydrate Antigen 19-9, and Carbohydrate Antigen 72-4 in Gastrointestinal Cancers. World J. Gastroenterol..

[44] Herrera-Pariente C., Montori S., Llach J., Bofill A., Albeniz E., Moreira L. (2021). Biomarkers for Gastric Cancer Screening and Early Diagnosis. Biomedicines.

[45] Xian X., Tang L., Wu C., Huang L. (2018). Mir-23b-3p and Mir-130a-5p Affect Cell Growth, Migration and Invasion by Targeting Cb1r Via the Wnt/Β-Catenin Signaling Pathway in Gastric Carcinoma. OncoTargets Ther..

[46] Figueroa-Protti L., Soto-Molinari R., Calderon-Osorno M., Mora J., Alpizar-Alpizar W. (2019). Gastric Cancer in the Era of Immune Checkpoint Blockade. J. Oncol..

[47] Giaquinto A.N., Miller K.D., Tossas K.Y., Winn R.A., Jemal A.M., Siegel R.L. (2022). Cancer statistics for African American/Black People 2022. CA Cancer J Clin..

